# Ingenol mebutate induces a tumor cell-directed inflammatory response and antimicrobial peptides thereby promoting rapid tumor destruction and wound healing

**DOI:** 10.1186/s40001-018-0343-8

**Published:** 2018-09-28

**Authors:** Stephan Alexander Braun, Julia Baran, Holger Schrumpf, Bettina Alexandra Buhren, Edwin Bölke, Bernhard Homey, Peter Arne Gerber

**Affiliations:** 1 Department of Dermatology, Medical Faculty, Heinrich Heine University, Düsseldorf, Germany; 2 Department of Orthopedics, Medical Faculty, Heinrich Heine University, Düsseldorf, Germany; 3 Department of Radiation Oncology, Medical Faculty, Heinrich Heine University, Düsseldorf, Germany

**Keywords:** Actinic keratosis, Anogenital warts, Chemokines, Condylomata acuminata, IL-8, Scratch assay

## Abstract

**Background:**

Ingenol mebutat (IM)-gel is effective for the topical treatment of epithelial tumors, including actinic keratoses (AKs) or anogenital warts (AGW). AK patients treated with IM develop intensified inflammatory reactions on sights of prior clinical visible or palpable AKs as compared to the surrounding actinically damaged skin, suggesting the induction of a tumor cell-directed inflammation. AGW patients treated with IM develop even stronger inflammatory reactions with large erosions, suggesting a directed inflammatory response against HPV-infected keratinocytes. Of note, even widespread erosions heal very fast without any superinfections. Here, we set out to elucidate underlying molecular and cellular mechanisms of these clinical observations.

**Methods:**

The effects of IM (10^−9^–10^−5^ M) on the expression and translation of a comprehensive set of chemokines (*CXCL1, CXCL8, CXCL9, CXCL10, CXCL11, CXCL14, CCL2, CCL5, CCL20, CCL27*) and antimicrobial peptides (AMP) (HBD1, HBD2, HBD3, LL37, RNase7) were analyzed in primary human epithelial keratinocytes (HEK) and a set of epithelial cancer cell lines by RT-qPCR and ELISA in vitro. To study the possible effects of different concentrations of IM on migratory, respectively wound healing responses, an in vitro scratch assay was conducted on HEK.

**Results:**

Ingenol mebutat significantly and dose-dependently induced the expression of proinflammatory chemokines (CXCL8, CCL2) and AMP (RNase7, HBD3) in HEK and epithelial cancer cell lines. A significantly stronger induction of CXCL8 and CCL2 was observed in our tested tumor cells as compared to HEK. We did not observe any significant effect of IM on HEK migration, respectively wound healing responses in vitro for any tested concentration (10^−9^, 10^−8^, 10^−6^ M) except 10^−7^ M, which induced a significant inhibition.

**Conclusions:**

Our data suggest that tumor cells are more susceptible to IM as compared to differentiated HEK. This is evident by a stronger IM-mediated induction of proinflammatory chemokines in tumor cells, which may result in a tumor cell-directed inflammatory response and rapid tumor destruction. In addition, IM induces AMP in keratinocytes and seems not to severely interfere with keratinocyte migration, which contributes to a fast and uncomplicated wound healing. Surprising is a selective inhibition of keratinocyte migration by IM at the concentration of 10^−7^ M pointing to very dose depending biological effects, induced by IM.

## Background

Ingenol mebutate (IM) is a macrocyclic diterpene ester, which is approved for the field therapy of actinic keratoses (AKs) [1]. Patients treated with IM develop a marked local inflammatory reaction with erythema, scaling, erosions and pustules with a peak on day 4 of the treatment [1]. The exact underlying mode of action of IM is still not completely understood, but a dual mechanism of action is proposed [2]: In high concentrations (~ 200–300 µM), IM induces direct necrotic and apoptotic cell death [3–5]; at low concentrations (~ 10^−7^ M), IM acts as a specific activator of protein kinase C (PKC) isoforms, leading to a release of proinflammatory cytokines and chemokines and activation of endothelial cells, resulting in the recruitment of a neutrophil-rich inflammatory infiltrate [6–10]. Li et al. [11] also demonstrated that IM is a substrate of P-glycoprotein, an ABC drug transporter, which facilitates dermal penetration of IM inducing vascular damage in a mouse model.

**Fig. 1:**
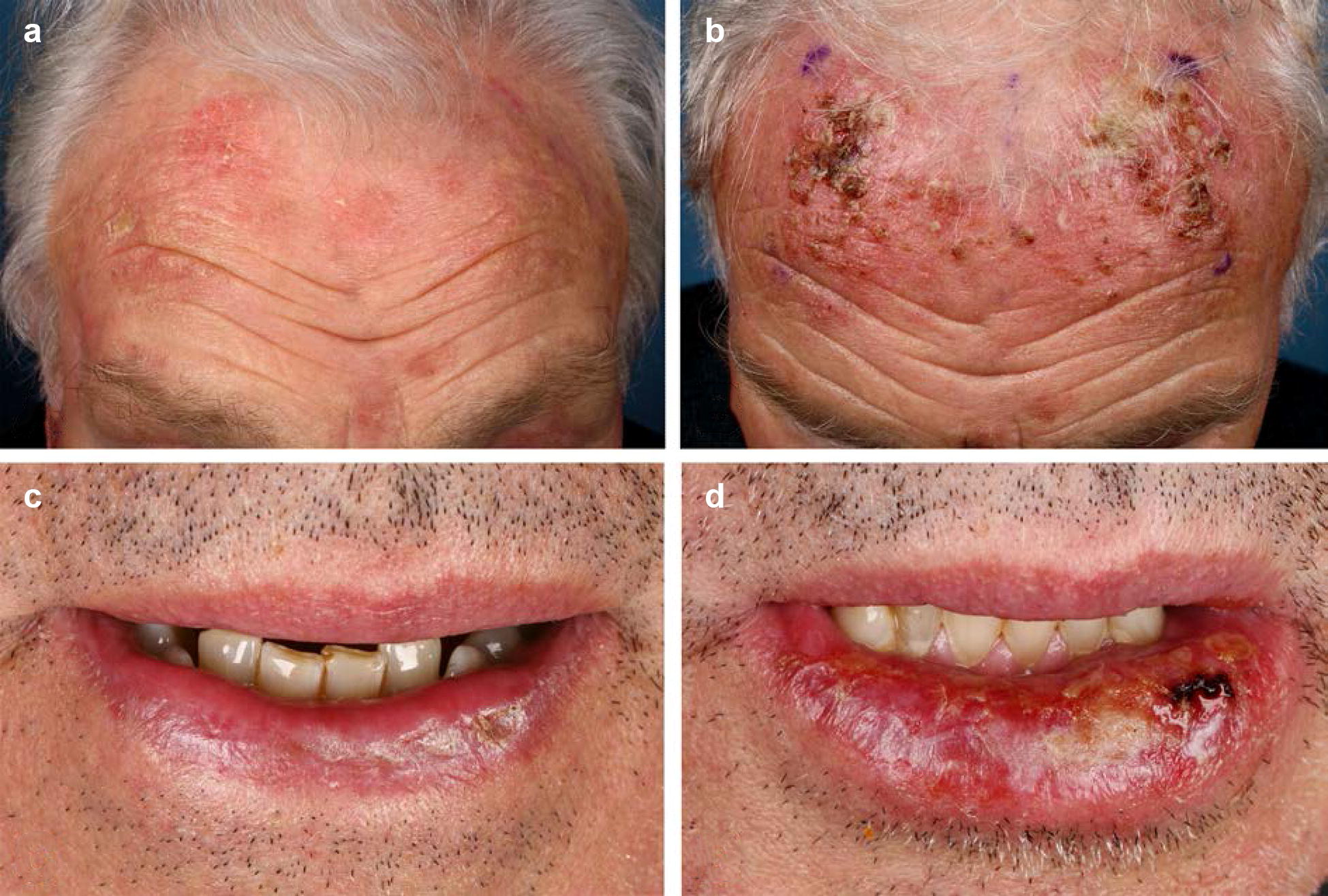
Intensified inflammatory reaction on sights of clinical visible actinic keratosis (AKs) compared chronic actinic damaged field on day 4 after a therapy with ingenol mebutate (IM)-0.015%-gel for 3 consecutive days. **a**, **b** Show an reaction on the forehead; **c**, **d** an example of an actinic cheilitis of the lower lip

**Fig. 2:**
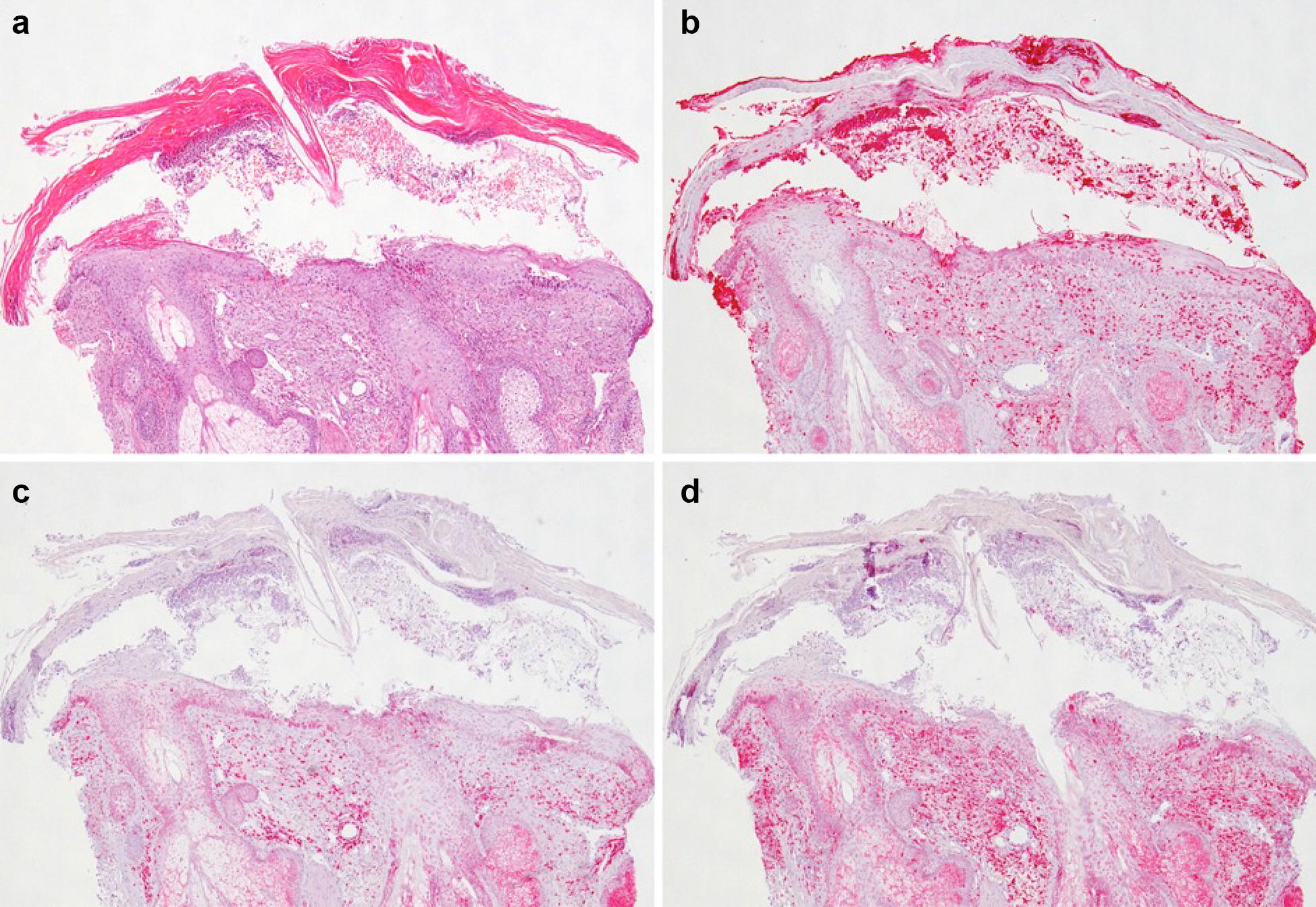
Ingenol mebutate (IM) induces a mixed inflammatory infiltrate. Histopathology of an actinic keratosis of the scalp on day 4 after a treatment with ingenol mebutate (IM)-0.015%-gel for 3 consecutive days. IM recruits a dense neutrophil-rich infiltrate (**a**) (hematoxylin and eosin stain). Myeloperoxidase staining reveals also abundant neutrophils in the *Stratum corneum*, forming microabscesses (**b**). CD3 stains for chemoattracted T cells (**c**), CD68 for histiocytes and neutrophils (**d**)

**Fig. 3:**
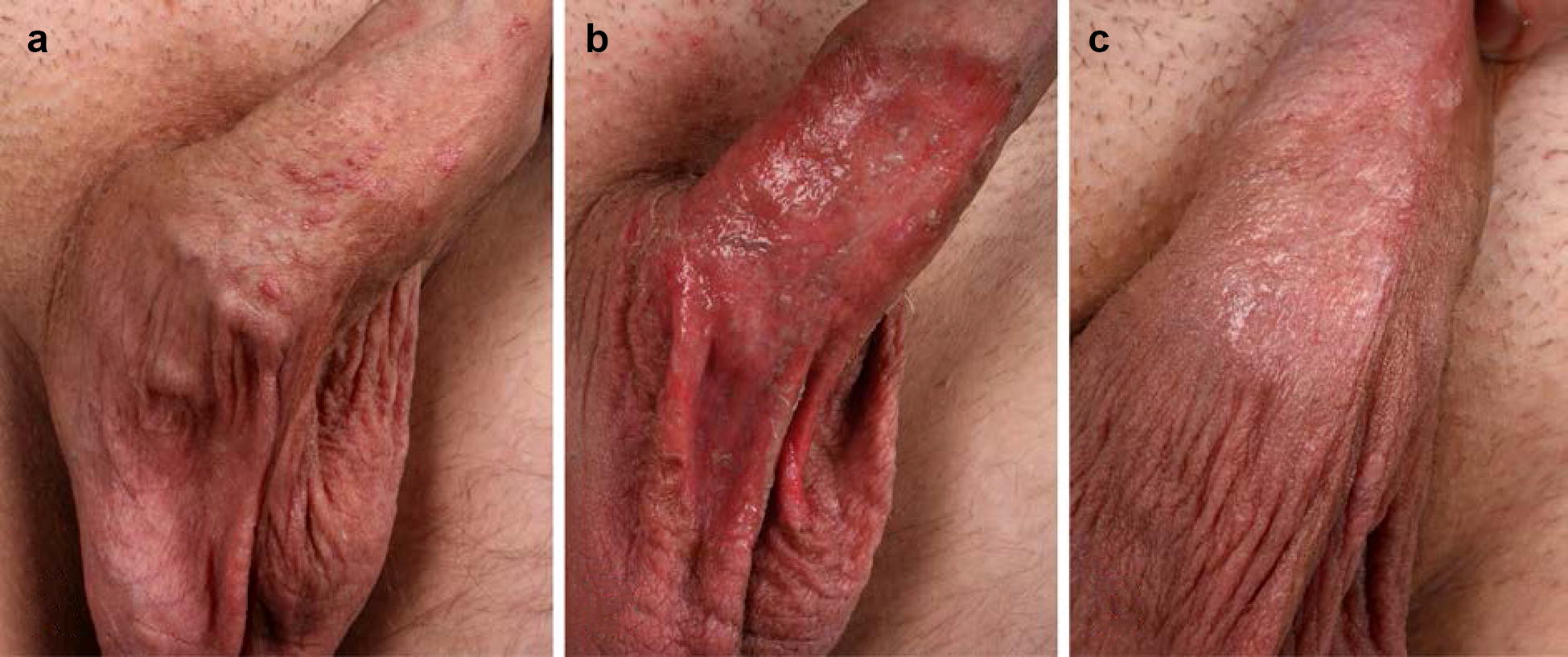
Uncomplicated wound healing of an extensive erosive dermatitis induced by a treatment of ingenol mebutate (IM)-0.05%-gel for anogenital warts (AGW). **a** Shows several AGW on the penile shaft. One drop of IM-gel was applied on all visible warts. Within 48 h, the patient developed an erosive dermatitis without any signs of superinfection in the treated area (**b**), which healed fast within the next 4 days with partial clearance of the AGW (**c**)

In recent years, we have made the clinical observation that patients with multiple AKs in chronic actinic damaged skin (actinic field cancerization), who have received a treatment with IM-gel, developed an intensified inflammatory reaction on sights of prior clinical visible or palpable AKs (Fig. 1). Representative histopathologic analyses of these AKs show that beside neutrophils, also T cells and dendritic cells are recruited to the treatment site (Fig. 2). A similar reaction accounts for anogenital warts (AGW) treated with IM [12–15]. Again, inflammatory reactions appear to be exaggerated in the areas of AGW as compared to surrounding, wart-free skin. Of note, patients with larger fields of AGW treated with IM may develop a widespread erosive dermatitis in the respective region (Fig. 3b). Against our expectations, we did not observe any clinical (increase of exudation, fibrinous crusts, erythema and pain) and microbiological (swabs without pathological germs) evidence for bacterial super infections of these erosive lesions. In addition, erosions appear to heal fast within a few days to a week [12–14].

To conclude, our clinical observations suggest that IM (i) induces a lesion-directed inflammatory reaction, (ii) sustains an effective antimicrobial defense despite a severe erosive barrier disruption and (iii) does not interfere with wound healing.

Here, we set out to elucidate underlying molecular and cellular mechanisms of our clinical observations. To this end, we have (i) analyzed the expression of a selected panel of proinflammatory chemokines in different tumor cell lines treated with IM in comparison to primary human keratinocytes, (ii) assessed the regulation of antimicrobial peptides, and (iii) analyzed in vitro wound healing reactions in respective keratinocytes.

## Methods

### Cell culture

Primary human cells and cancer cell lines were cultured as previously defined [16, 17]. Primary human epithelial keratinocytes (HEK) and epithelial cancer cell lines (UD-SCC 8, UD-SCC 1, UD-SCC 2, UD-SCC 7A) were cultured in Dulbecco’s Modified Eagle Medium (DMEM; ThermoFisher Scientific, Waltham, USA) supplemented with 10% fetal calf serum, 1% sodium pyruvate (Merck Millipore, Billerica, USA), 1% l-glutamine, and 1% penicillin–streptomycin. Cells of HeLa line were grown in Minimum Essential Medium (MEM; PAA, Pasching, Austria) with addition of 10% fetal calf serum. All cells were incubated at 95% humidity, 5% CO_2_ and 37 °C. Subsequently, cells were treated with different concentrations (10^−9^, 10^−8^, 10^−7^, 10^−6^, 10^−5^ M) of ingenol mebutate (IM; LEO Pharma, Ballerup, Denmark) or in medium as control.

### Quantitative real-time PCR analysis

Quantitative real-time PCR (RT-qPCR) analyses were performed as described previously [18]. Total RNA from cultured cells was isolated for expression analysis using RNeasy Mini Kit (Qiagen, Hilden, Germany). cDNA synthesis was realized with SuperScript first strand synthesis system (ThermoFisher Scientific, Waltham, USA) according to the manufacturer’s instructions. qRT-PCR was performed in the presence of SYBR Green master mix (ThermoFisher, Waltham, MA). Gene expression levels of different chemokine ligands (CXCL1, CXCL8, CXCL9, CXCL10, CXCL11, CXCL14, CCL2, CCL5, CCL20, CCL27) and antimicrobial peptides (HBD1, HBD2, HBD3, LL37, RNase7) were measured after 24 h incubation of IM. All primers used were provided by Eurofins MWG (Ebersberg, Germany). Target gene expression was normalized to the expression of 18S rRNA. Gene-specific PCR products were detected by means of a QuantStudio 6 Flex Real-Time PCR system (ThermoFisher Scientific, Waltham, USA).

### Enzyme-linked immunosorbent assay

Furthermore, quantification of CXCL8 protein concentrations in cell culture supernatants collected from 80% confluent cells was performed by enzyme-linked immunosorbent assay (ELISA) according to DuoSet ELISA development kit protocol (R&D Systems, Minneapolis, USA). Optical density at a wavelength of 450 nm was recorded in an ELISA reader (Multiskan Ascent, ThermoFisher Scientific, Waltham, USA).

### Wound healing assay

A monolayer of HEK cells was scratched using a 10 μl pipette tip (Starlab, Hamburg, Germany) in order to create a 1 mm cell-free area. Cells were then stimulated with IM (see above). This was set as time point zero. At 100× magnification, photo documentation (Zeiss Axiovert 200 M and Axiovision software v4.7, Oberkochen, Germany) of the cell movements was done. Shots were recorded at an interval of 5 min over a period of 40 h. During this time, a temperature of 37 °C and a CO_2_ concentration of 5% were ensured. Evaluation of the data was carried out with TScratch software (v1.0, ETH Zurich, Switzerland).

### Statistical analysis

For statistical analysis, GraphPad Prism software (v5.03 GraphPad Software, La Jolla, USA) was used. Data are represented as mean ± standard error of the mean (SEM). *n* describes the number of biological replicates. Data were evaluated with a two-tailed, unpaired Student’s *t* test with 95% confidence intervals. *p* ≤ 0.05 was taken to be statistically significant, and *p* values are represented with asterisks in the figures (*p ≤ 0.05; **p ≤ 0.01; ***p ≤ 0.001).

## Results

### Ingenol mebutate super-induces the expression of CXCL8 and CCL2 in epithelial tumor cell lines as compared to human epithelial keratinocytes

To analyze whether the different intensities of inflammation in AKs versus the surrounding actinic damaged skin correlate with different expression levels of proinflammatory chemokines, in tumor cells, we measured the expression levels of a selected panel of chemokines in squamous cancer cell lines and compared the levels with those of non-neoplastic human epithelial keratinocytes (HEK) incubated with IM (10^−9^, 10^−8^, 10^−7^, 10^−6^, 10^−5^ M) or medium control.

**Fig. 4:**
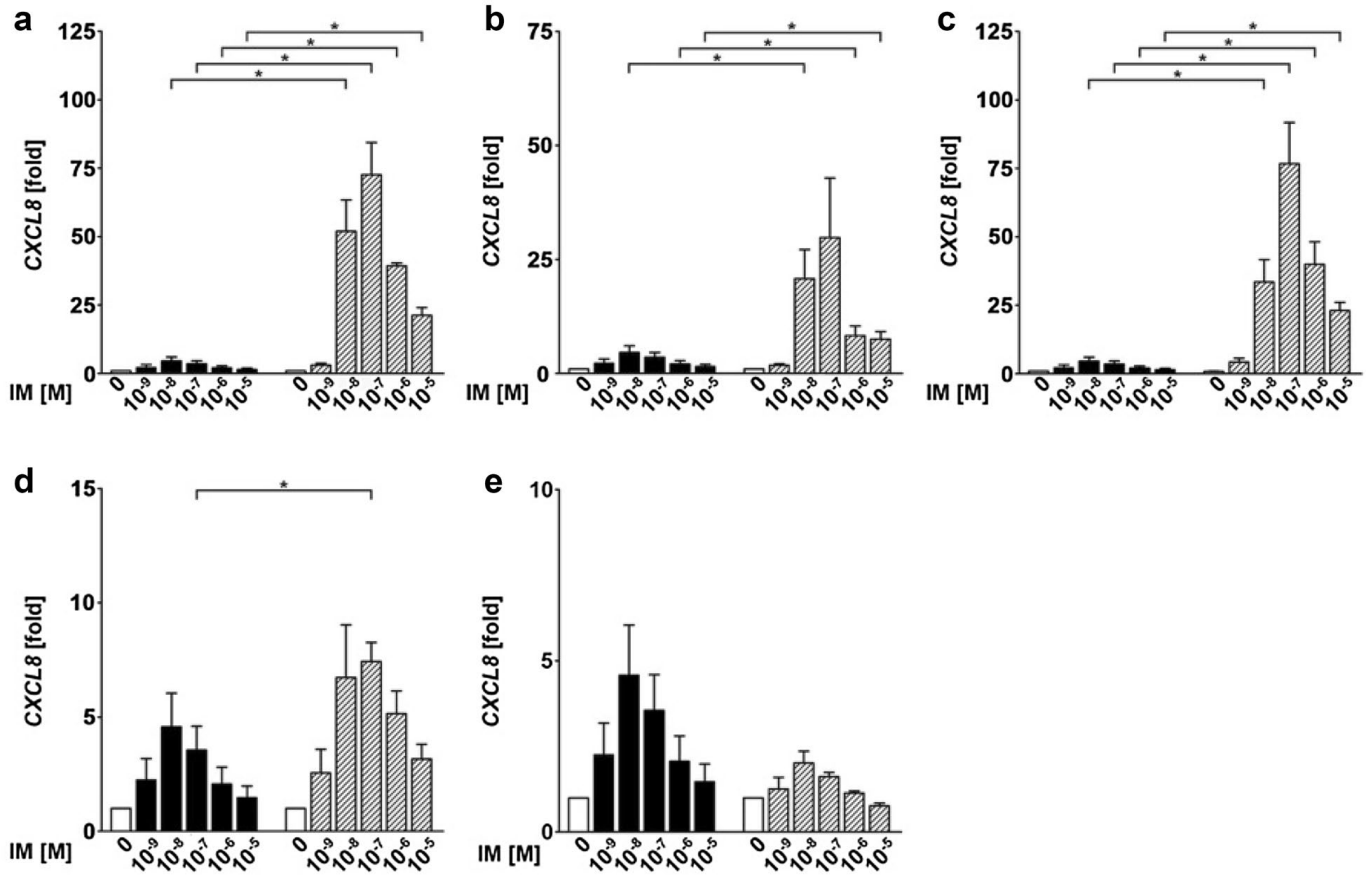
CXCL8 is super-induced in most of the tested squamous cancer cell lines compared to human epithelial keratinocytes (HEK). The graphs show the expression of CXCL8 in HEK (black bars) compared to the cancer cell lines (gray bars) UD-SCC 8 (**a**), HeLa (**b**), UD-SCC 1 (**c**), UD-SCC 2 (**d**) and UD-SCC 7A (**e**). Significances were calculated by an unpaired Student’s *t* test (*p < 0.05, **p < 0.01 and ***p < 0.001); *IM* ingenol mebutate, *M* molar

**Fig. 5:**
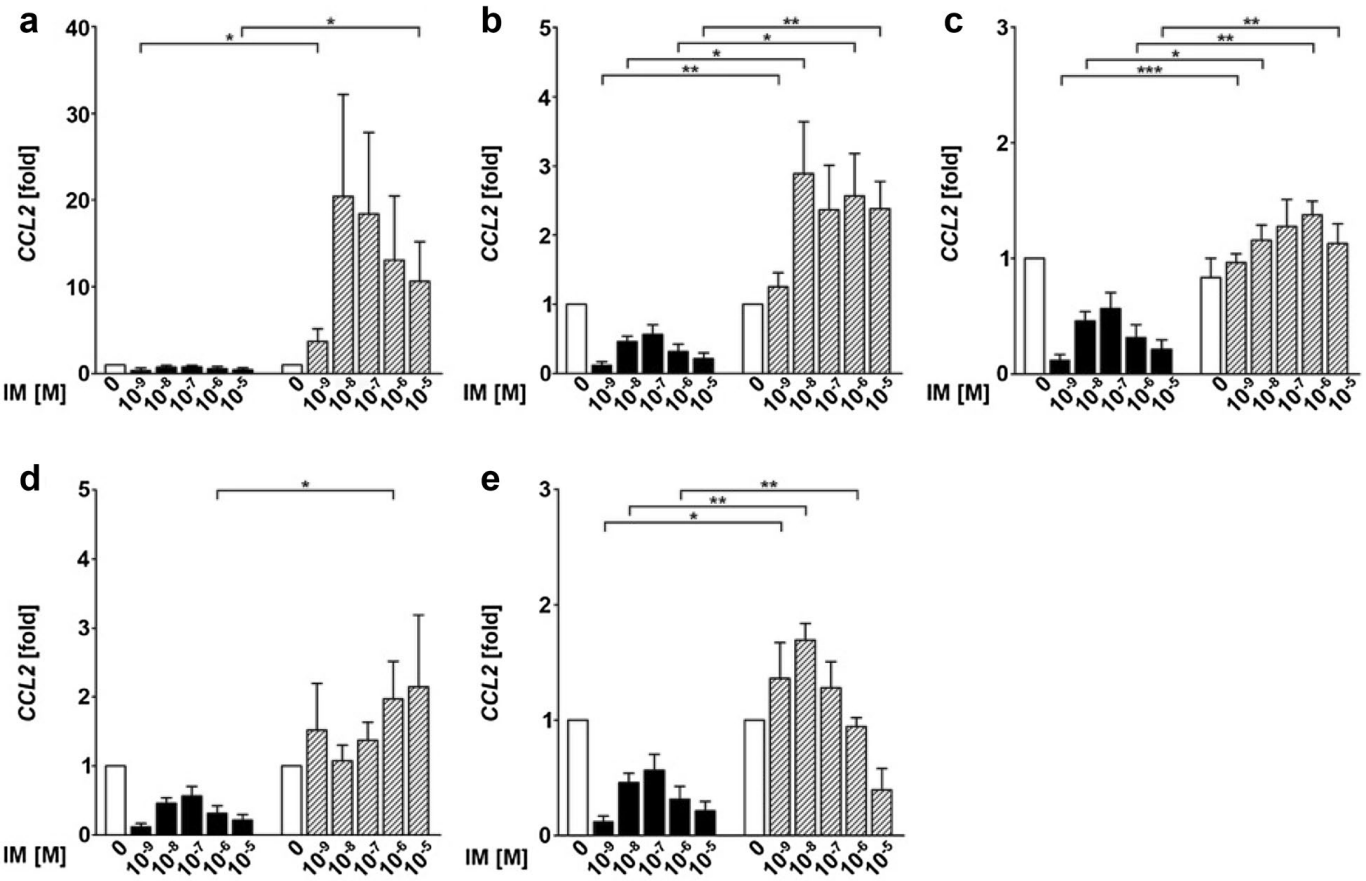
CCL2 is up regulated in squamous cancer cell lines compared to human epithelial keratinocytes (HEK). The graphs show the expression of CCL2 in HEK (black bars) compared to the cancer cell lines (grey bars) UD-SCC 8 (**a**), HeLa (**b**), UD-SCC 1 (**c**), UD-SCC 2 (**d**) and UD-SCC 7A (**e**). Significances were calculated by an unpaired Student’s *t* test (*p < 0.05, **p < 0.01 and ***p < 0.001); *IM* ingenol mebutate, *M* molar

The strongest and most consistent induced chemokine of the investigated panel was CXCL8 (IL-8). CXLC8 was both induced by IM in HEK and neoplastic tumor cell lines (UD-SCC 8, UD-SCC 1, UD-SCC 2, UD-SCC 7A, HeLa-cells; Fig. 4a–e). The expression levels were inconsistent between the different cell lines with the highest expression seen in the squamous cancer cell line UD-SCC 8 (Fig. 4b) and the lowest in UD-SCC 7A (Fig. 4e). They were also dependent on the concentration of IM, showing the strongest induction between 10^−8^ and 10^−7^ M. All tumor cell lines, but UD-SCC 7A, showed significantly higher expression levels of CXCL8 compared to HEK for most of the used concentrations (Fig. 4a–e). For CCL2, there was no significant regulation in both HEK and the investigated cancer cell lines (Fig. 5a–e). However, again, in comparison to HEK, the expression of CCL2 in the cancer cell lines UD-SCC 8, UD-SCC 2, UD-SCC 1 and HeLa was significantly upregulated for most of the concentrations tested (Fig. 5a–d). Additional qRT-PCR-analysis of the chemokines CXCL1, CXCL9, CXCL10, CXCL11, CXCL14, CCL20 and CCL27 did not show any consistent regulation of expression after stimulation with IM in any concentration in both tumor cell lines and HEK (data not shown).

### Higher expression levels of CXCL8 in the tumor cell lines UD-SCC 8 and HeLa correlate with higher CXCL8 protein secretion

**Fig. 6:**
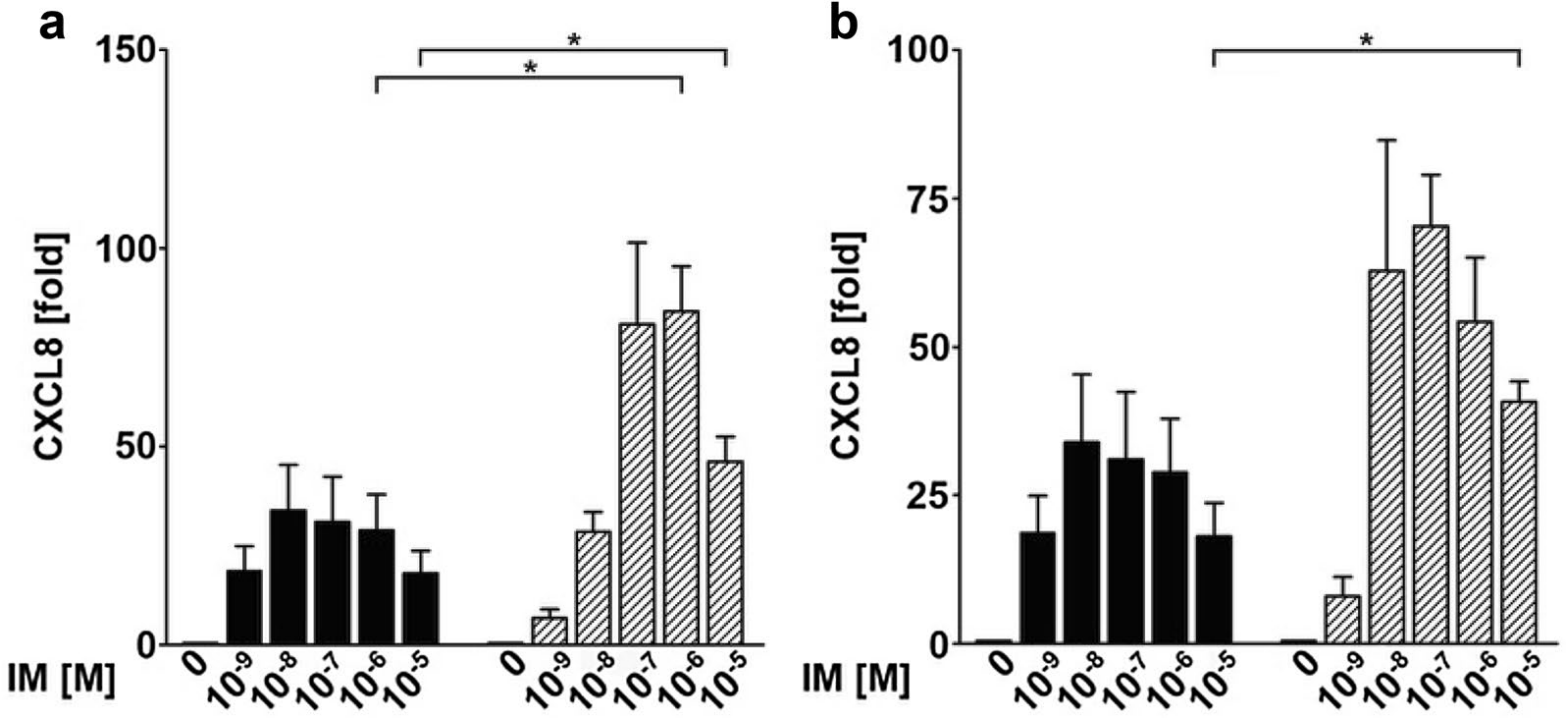
Higher expression levels of CXCL8 in the tumor cell lines correlate with higher CXCL8 protein secretion in supernatants compared to human epithelial keratinocytes (HEK). The graphs show the protein level measured by ELISA of CXCL8 in HEK (black bars) compared to the cancer cell lines (grey bars) UD-SCC 8 (**a**), HeLa (**b**). Significances were calculated by an unpaired Student’s *t* test (*p < 0.05, **p < 0.01 and ***p < 0.001); *IM* ingenol mebutate, *M* molar

Since CXCL8 was the chemokine most consistently induced by IM, we next assessed CXCL8 protein concentrations in conditioned media of the tumor cell lines UD-SCC 8 and HeLa treated with IM or medium controls by ELISA. Significantly higher levels of CXCL8 were detected in supernatants of tumor cell lines treated with IM as compared to supernatants of primary human epithelial keratinocytes (Fig. 6).

### Ingenol mebutate induces the expression of antimicrobial peptides in primary human keratinocytes

**Fig. 7:**
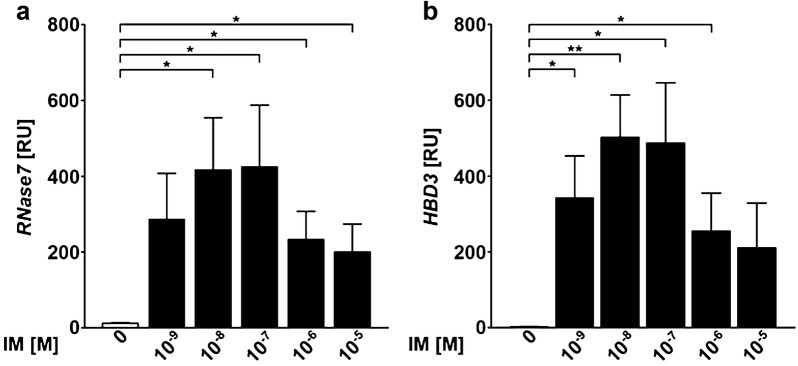
Ingenol mebutate induces the expression of antimicrobial peptides RNase7 and HBD3 in primary human keratinocytes. Significances were calculated by an unpaired Student’s *t* test (*p < 0.05, **p < 0.01 and ***p < 0.001); *IM* ingenol mebutate, *M* molar

The relative resistance of even widespread skin erosions induced by topically applied IM against bacterial superinfections suggests an IM-mediated induction of antimicrobial mediators. To test this hypothesis, we assessed an induction of antimicrobial peptides (AMP) by IM in primary human keratinocytes in vitro by qRT-PCR. Indeed, IM induced a significant expression of Ribonuclease A Family Member 7 (RNase7) and human beta-defensin 3 (HBD3) (Fig. 7). No regulation was observed for HBD1, HBD2 and LL37/cathelicidine (data not shown).

### Ingenol mebutate interferes with keratinocytes migration in a dose-dependent manner

**Fig. 8:**
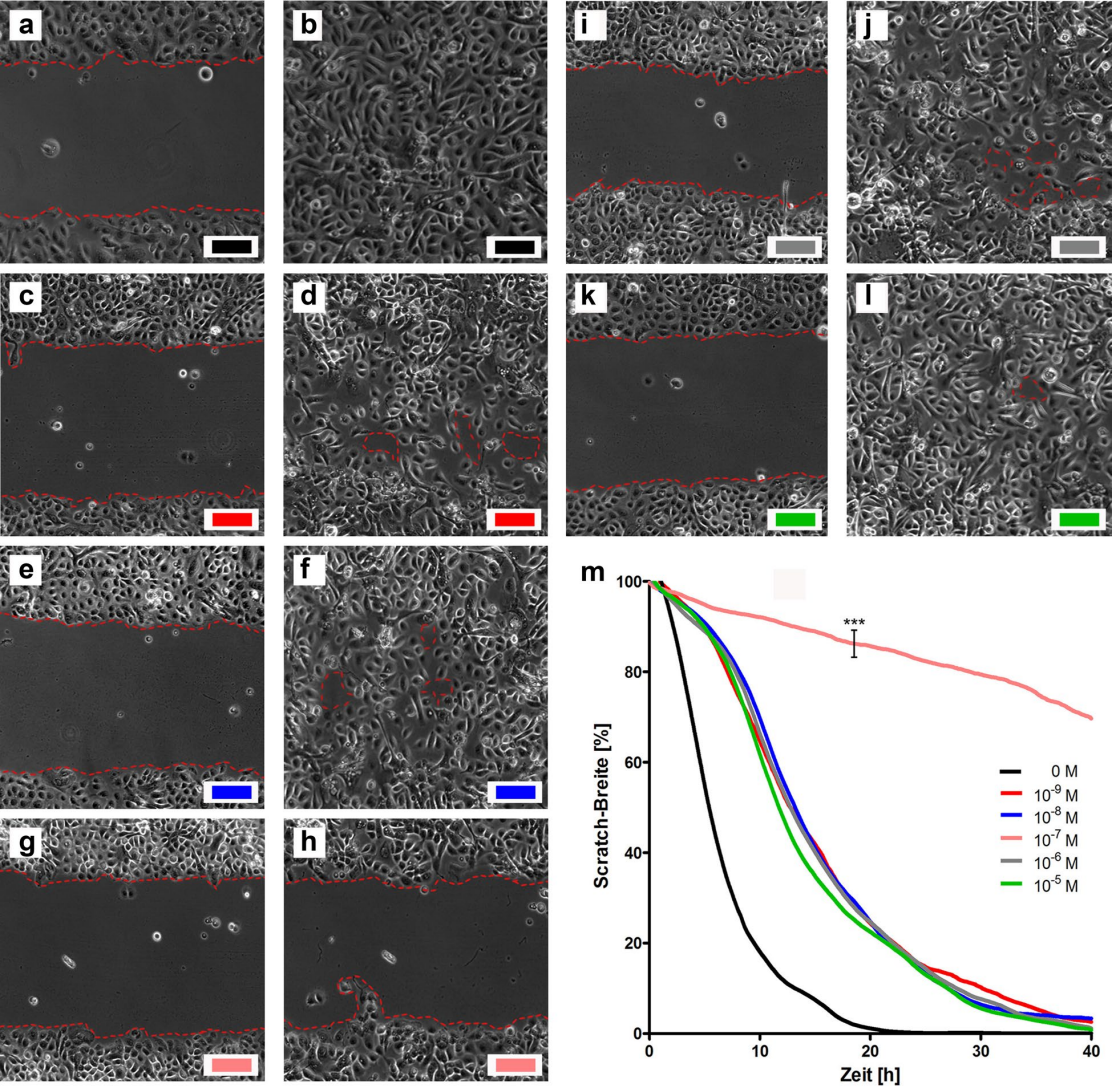
Ingenol mebutate interferes with human epithelial keratinocytes (HEK) migration in a dose-dependent manner. HEK migration was studied in a scratch-assay stimulated by different concentrations of ingenol mebutate (IM). The picture pairs (**a**–**b**; **c**–**d**; **e**–**f**; **g**–**h**; **i**–**j**; **k**–**l**) show the migration of the HEK for the time-points *t* = 0 h and *t* = 22 h for different concentrations of IM (colors match concentrations in graph **m**). **m** Shows the relative scratch width of three independent assays over 40 h for different concentrations. Interestingly, IM applied in a concentration of 10^−7^ significantly inhibited cell migration, whereas all other concentrations did not influence keratinocyte migration. Significances were calculated by an unpaired Student’s *t* test (*p < 0.05, **p < 0.01 and ***p < 0.001); *M* molar

To elucidate whether IM influences cutaneous wound healing, we performed scratch-wound assays with HEK. The assays show that IM does not significantly interfere with keratinocyte migration in most of the tested concentrations (10^−9^, 10^−8^, 10^−6^, 10^−7^ M). However, at a concentration of 10^−7^ M IM significantly inhibited cell migration (Fig. 8).

## Discussion

Clinical observations suggest that IM induces a lesion-directed immune response that significantly contributes to the clinical efficacy of the drug against epithelial tumors. This hypothesis is supported by representative histopathologic analyses of AKs treated with IM that show the recruitment of a mixed inflammatory infiltrate of neutrophils, T cells and histiocytes directly from the vessels to the epidermal lesions (Fig. 2).

Here, we demonstrate that in epithelial derived cells IM induces a marked expression of the proinflammatory chemokines CXCL8 and CCL2 that subsequently can recruit different subsets of immune cells. Out of all tested chemokines, CXCL8 (IL-8) is the only chemokine, which was consistently induced by IM in both human epidermal keratinocytes and most of the epithelial tumor cell lines. This is in agreement with previously published data, showing that IM upregulates CXCL8 in keratinocytes, fibroblast, neutrophils and melanoma cells in vitro as well as MIB-2, the murine homologue of CXCL8, in IM-treated mouse skin [6]. CXCL8 was also upregulated in human skin biopsies of AKs treated with IM [7]. Strikingly, in our analyses we could demonstrate that in neoplastic cell lines the induction of CXCL8 is higher as compared to unaltered primary cells. In all but one tumor cell line we could demonstrate a significant up to 75-fold higher expression of CXCL8 as compared to HEK. CXCL8 is a strong attractant for neutrophils and, therefore, explains the recruitment of the neutrophil-rich infiltrate, which is characteristic for IM and clinically correlates to the formation of pustules (Fig. 1b, d) [19]. Since we expect more neoplastic cells in an area of clinical visible actinic keratoses, the superinduction of CXCL8 in neoplastic cancer cells could explain the stronger inflammatory reaction in these areas. Moreover, we observed a moderate, yet not significant, induction of CCL2 in most of our tumor cell lines. CCL2 is a chemoattractant for monocytes and T cells [20]. Accordingly, the IM-mediated induction of CCL2 may explain the recruitment of T cells and macrophages to the treatment area, which is in line with our histopathology and results obtained by Emmert et al. [7], who also describe a mixed inflammatory infiltrate in histology of AK treated with IM.

Yet, none of our tested chemokines showed a consistently significant regulation in all tested cell lines, which could correlate to the late time point for our PCR analysis performed after 24 h. This hypothesis is supported by the fact that we detected a significant higher production of CXCL8 protein in our ELISA in conditioned supernatants of UD-SCC8 cells after 24 h. Conceivably after 24 h most of the mRNA has already been translated or depleted. Another limitation of our investigation is the choice tumor cell lines. Although squamous cancer cell lines from different sites share abundant molecular markers [21], squamous cell carcinoma cell lines from the skin may have represented the “actinic keratosis tumor cell” better than cell lines from head and neck and cervical cancer.

With regard to the relative resistance of IM-induced skin erosions against bacterial superinfections, we could show that IM induces the expression of the antimicrobial peptides (AMP) RNase7 and HBD3. AMP play a critical role in warding off microbial pathogens and also improve wound healing [22]. HBD3 and RNase7 show strong antimicrobial activity against a broad spectrum of microbes including *Staphylococcus aureus* and even vancomycin-resistant *Enterococcus faecium* and *Candida albicans* [23, 24]. It has also been shown that HBD3 stimulates keratinocyte migration and proliferation [25]. Therefore, although AMP are already upregulated in HPV-infected lesions [26], the additional induction of AMP in keratinocytes in addition to the recruitment of neutrophils seems to contribute to the uncomplicated course of wound healing after a treatment of genital warts with IM-gel in our patients.

The results of our wound healing assay suggest that IM does not significantly distract the migration of keratinocytes in most of the tested concentrations and, therefore, does not negatively interfere with wound healing. This is again in line with the clinical observation, that erosive wounds, induced by topically applied IM-gel, heal fast and uncomplicated within several days. Interestingly, IM at a concentration of 10^−7^ M did, however, significantly inhibited keratinocyte migration. A systematic analysis of our gene expression data revealed that IM at concentrations between 10^−8^ and 10^−7^ M also showed the strongest induction of our tested chemokines and antimicrobial peptides, whereas higher and lower concentrations of IM-induced lower expression rates, resulting in a bell-shaped pattern of gene expression. It is known that in these low, non-cytotoxic concentrations, IM primarily mediates its activity by binding to protein kinase C (PKC) isoforms and signals via PKC/MEK/ERK in keratinocytes [10, 27]. Since it has been shown that PKC mediates proinflammatory cytokine and chemokine production and inhibits keratinocyte migration [10, 28, 29], we suggest that firstly the production of proinflammatory chemokines in our cell lines and the migration of keratinocytes are also mediated by a specific activation of PKC isoforms by IM, and that secondly this activation or interaction is very dose dependent, inducing the greatest biologic effect at concentrations around 10^−7^ M.

If you compare IM to other, already longtime established therapeutic agents for AKs and genital warts, our and the already published data indicate that IM combines important modes of action in the treatment of non-melanoma skin cancer (NMSC) and HPV-induced warts. Firstly, IM acts as a cytotoxic agent inducing necrosis and apoptosis of tumor or viral-infected cells, comparable to fluorouracil (5-FU) or podophyllotoxin. Secondly, IM develops anti-tumor effects by the induction of an inflammatory infiltrate and therefore acts as an ‘immune response modifier’. Now, beyond that, our data indicate that IM also acts with lesion-directed properties comparable to imiquimod or photodynamic therapy (PDT) [30, 31]. From a clinical point of view, a lesion-preferential induction of inflammatory reactions is of particular interest, since side effects in the not affected treatment area can be reduced. For both imiquimod and PDT also the induction of systemic immunologic effects has been shown [30–33]. In line with these observations, a systemic immune reaction with the formation of specific T cells against, e.g. HPV antigens by IM would be of great interest in the treatment of AGW, since this might reduce the recurrence rate and induce reactions on subclinical sites, as it has already been shown for imiquimod [34]. However, up-to-date there is only a few evidence in the literature that IM also induces T cell-mediated systemic anti-tumor effects. Therefore, further clinical in vivo studies are urgently needed to elucidate the effect of IM on NMSC, AGW and potentially other epithelial tumors.

## Conclusions

The results presented in this study suggest that genetically altered epithelial tumor cells are more susceptible to IM as compared to differentiated human keratinocytes, resulting in a stronger induction of proinflammatory chemokines in tumor cells, pointing towards a tumor cell-directed mode of action. In addition, IM upregulates AMP and does not interfere with keratinocyte migration in most concentrations, which contributes to a fast and uncomplicated wound healing. Worth of note, wounding- or cell migration analysis illustrates that IM acts in a very dose-dependent manner and can even induce biological ‘on–off phenomenas’ by choosing specific concentrations. Future experiments should assess the influence of different expression levels and phosphorylation patterns of PKC isoforms in the tumor cell lines, of different concentrations of IM, and the kinetics of IM-induced effects. Furthermore, future in vivo studies may investigate potential systemic immune-stimulatory effects induced by locally applied IM.

## Abbreviations

ABC: ATP-binding cassette
AGW: anogenital warts
AK: actinic keratosis
AMP: antimicrobial peptides
CD: cluster of differentiation
ELISA: enzyme-linked immunosorbent assay
ERK: extracellular signal-regulated kinase
HBD: human beta-defensin
HE: hematoxylin and eosin (staining)
HEK: human epithelial keratinocytes
HPV: human papilloma virus
IM: ingenol mebutate
MEK: mitogen-activated protein kinase
MPO: myeloperoxidase (staining)
PDT: photodynamic therapy
PKC: protein kinase C
RT-qPCR: real time quantitative polymerase chain reaction
RNase7: Ribonuclease A Family Member 7
SCC: squamous cancer cell
SEM: standard error of the mean
